# Visible-Light-Responsive Ag(Au)/MoS_2_-TiO_2_ Inverse Opals: Synergistic Plasmonic, Photonic, and Charge Transfer Effects for Photoelectrocatalytic Water Remediation

**DOI:** 10.3390/nano15141076

**Published:** 2025-07-11

**Authors:** Stelios Loukopoulos, Elias Sakellis, Polychronis Tsipas, Spiros Gardelis, Vassilis Psycharis, Marios G. Kostakis, Nikolaos S. Thomaidis, Vlassis Likodimos

**Affiliations:** 1 Section of Condensed Matter Physics, Department of Physics, National and Kapodistrian University of Athens, University Campus, 15784 Athens, Greece; sloukop@phys.uoa.gr (S.L.); e_sakel@phys.uoa.gr (E.S.); sgardelis@phys.uoa.gr (S.G.); 2 Institute of Nanoscience and Nanotechnology, National Center for Scientific Research “Demokritos”, Agia Paraskevi, 15341 Athens, Greece; p.tsipas@inn.demokritos.gr (P.T.); v.psychairs@inn.demokritos.gr (V.P.); 3 Laboratory of Analytical Chemistry, Department of Chemistry, National and Kapodistrian University of Athens, University Campus, 15771 Athens, Greece; makostak@chem.uoa.gr (M.G.K.); ntho@chem.uoa.gr (N.S.T.)

**Keywords:** titanium dioxide, inverse opal, molybdenum disulfide, plasmonic photocatalysis, localized surface plasmon resonance, slow photon, visible-light activation, photoelectrocatalysis, tetracycline degradation

## Abstract

Titanium dioxide (TiO_2_) is a benchmark photocatalyst for environmental applications, but its limited visible-light activity due to a wide band gap and fast charge recombination restricts its practical efficiency. This study presents the development of heterostructured Ag (Au)/MoS_2_-TiO_2_ inverse opal (IO) films that synergistically integrate photonic, plasmonic, and semiconducting functionalities to overcome these limitations. The materials were synthesized via a one-step evaporation-induced co-assembly approach, embedding MoS_2_ nanosheets and plasmonic nanoparticles (Ag or Au) within a nanocrystalline TiO_2_ photonic framework. The inverse opal architecture enhances light harvesting through slow-photon effects, while MoS_2_ and plasmonic nanoparticles improve visible-light absorption and charge separation. By tuning the template sphere size, the photonic band gap was aligned with the TiO_2_-MoS_2_ absorption edge and the localized surface plasmon resonance of Ag, enabling optimal spectral overlap. The corresponding Ag/MoS_2_-TiO_2_ photonic films exhibited superior photocatalytic and photoelectrocatalytic degradation of tetracycline under visible light. Ultraviolet photoelectron spectroscopy and Mott–Schottky analysis confirmed favorable band alignment and Fermi level shifts that facilitate interfacial charge transfer. These results highlight the potential of integrated photonic–plasmonic-semiconductor architectures for efficient solar-driven water treatment.

## 1. Introduction

Titanium dioxide (TiO_2_) nanomaterials have been long investigated as benchmark semiconductor photocatalysts due to their high redox potential, chemical stability, and low cost [1,2]. In addition, TiO_2_ is largely considered non-toxic, though recent studies have raised concerns on the toxicity of TiO_2_ nanoparticles (NPs) to human health and the environment [3,4], indicating that their immobilization on suitable supports, which prevents material leaching, is the safest approach towards environmentally benign TiO_2_ photocatalytic nanomaterials [5]. However, their practical use in environmental remediation is limited by their wide band gap (3.0–3.2 eV) and low quantum efficiency, which depend critically on the structural, electronic, and morphological properties of TiO_2_, i.e., the nanomaterial phase, crystallinity, defect content, texture, size, and shape that largely impact charge carrier recombination and the process efficiency [6,7]. To overcome these limitations, heterojunction engineering, particularly with narrow-band-gap semiconductors and suitable co-catalysts, has emerged as an effective strategy to enhance visible-light absorption, promote charge separation, and facilitate surface redox reactions [8,9] for the abatement of contaminants of emerging concern by means of photocatalysis [10,11] and photoelectrocatalysis [12,13].

Molybdenum disulfide (MoS_2_), the prototype transition metal dichalcogenide, has emerged as a promising co-catalyst for TiO_2_ due to its distinctive electronic properties [14,15]. A key advantage of MoS_2_ lies in the tunability of its band gap, from 1.3 (indirect) to 1.9 eV (direct), which can be modulated by reducing the number of nanosheets (NSs) from bulk to monolayer levels, as a result of weak van der Waals interlayer coupling [16]. This band gap tunability, together with MoS_2_’s exceptional optoelectronic and mechanical characteristics, has expanded its application potential to hydrogen evolution and water remediation [17,18,19]. Despite these advantages, the performance of MoS_2_ photocatalysts is hindered by its low oxidation potential for hydroxyl radical formation and the limited number of active sites on its inert basal planes [20]. To circumvent these drawbacks, heterojunctions with nanostructured TiO_2_ supports have been extensively explored [21,22,23,24,25] in order to enhance visible-light absorption and facilitate charge separation for photocatalytic organics degradation.

Among various architectures, inverse opal (IO) structures stand out due to their three-dimensional photonic crystal (PC) framework [26,27], which enables slow-photon effects at the photonic band gap (PBG) edges. The overlap of slow photons with spectral regions of weak materials’ electronic absorption offers a unique structural approach for improving light harvesting of photocatalytic nanomaterials, especially TiO_2_ photocatalysts [28,29,30]. Additionally, the IO morphology offers a hierarchical porous structure with interconnected macropores and mesoporous skeletal walls [31], facilitating mass transport and pollutant adsorption that can be further combined with compositional and morphological modifications for visible-light-activated TiO_2_-based PC catalysts [32,33,34,35,36]. The co-assembly of templating colloids with metal oxide precursors has emerged as an advanced strategy that integrates template self-assembly and liquid-phase infiltration into a single-step process, leading to the fabrication of high-quality IO photocatalytic films [37,38]. This approach was further enhanced by incorporating noble metal NPs as a third component in the co-assembly mixture, enabling the creation of composite IO structures with plasmonic functionality [39,40,41]. More recently, a three-phase co-assembly method involving polymer colloids and water-soluble metal oxide precursors enabled the fabrication of compositionally tunable WO_3_/TiO_2_ IOs, featuring uniformly distributed nanoscale type II heterojunctions within the skeletal walls [42]. This single-step method was also successfully applied to incorporate MoS_2_ NSs into the nanocrystalline framework of TiO_2_ IOs, resulting in highly active MoS_2_–TiO_2_ photocatalysts for the visible-light degradation of pharmaceutical contaminants [43].

Plasmonic-assisted photocatalysis via metal–semiconductor heterojunctions has emerged as a powerful strategy to evade the limitations of poor light harvesting and inefficient charge separation in wide-band-gap semiconductors like TiO_2_ [44]. Beyond their long-established role as electron scavengers that suppress electron–hole recombination [9], metal co-catalysts such as Au and Ag NPs can enhance photocatalytic activity through both radiative and non-radiative decay of their localized surface plasmon resonance (LSPR) [45,46]. Radiative decay can induce strong near-field enhancement, significantly increasing electron–hole pair generation in adjacent semiconductors [47]. For larger NPs (typically >50 nm), light scattering becomes dominant [44,45], extending the photon path length and promoting further charge carrier generation. However, such enhancements are spectrally confined to the overlap between the semiconductor band gap and LSPR absorption. Non-radiative LSPR decay can lead to hot electron injection over the Schottky barrier at the metal–semiconductor interface [48,49] or to plasmon-induced resonant energy transfer [50], both of which enable visible-light activation of TiO_2_ without direct band gap excitation. Efforts to align the LSPR of Au and Ag NPs with slow-photon regions in photonic TiO_2_ structures have aimed to synergistically combine plasmonic and photonic effects for improved light harvesting [51]. Enhanced photocatalytic performance of PBG engineered IOs has been largely attributed to LSPR excitation facilitated by slow photons and efficient hot electron injection into the TiO_2_ conduction band [52,53,54,55,56]. More recently, electron scavenging due to interfacial band alignment and radiative near-field enhancement by plasmonic NPs have been identified as key factors in the improved (photo)electrocatalytic performance of plasmonic modified IOs [57,58,59].

In this work, heterostructured Ag(Au)/MoS_2_–TiO_2_ IO films were developed and investigated as photoelectrocatalysts for the degradation of tetracycline (TC), a broad-spectrum antibiotic of significant environmental concern [10]. The plasmonic–photonic structures were fabricated via the evaporation-induced self-assembly of sacrificial colloidal spheres with a water-soluble Ti precursor and aqueous dispersions of Ag(Au) NPs and MoS_2_ NSs. This approach enabled their effective incorporation into the nanocrystalline TiO_2_ IO framework and the formation of abundant, stable heterojunctions. Two distinct colloidal template sizes were employed to tune the slow-photon spectral regions to the TiO_2_ absorption edge and the visible absorption of MoS_2_, respectively, in order to explore the optimal amplification spectral range. The incorporation of MoS_2_ NSs and Ag NPs into the TiO_2_ IO structure led to a marked enhancement in both photocatalytic and photoelectrocatalytic activity under visible light. The best performance in TC degradation was achieved with Ag/MoS_2_–TiO_2_ IO films, where the red-shifted slow photons matched both the Ag LSPR absorption and the MoS_2_–TiO_2_ absorption edge, assisted by electron transfer from MoS_2_ NSs and Ag NPs acting as visible light sensitizers of TiO_2_ along with the local electromagnetic field enhancement at the metal–semiconductor interface due to LSPR.

## 2. Materials and Methods

### 2.1. Chemicals and Reagents

Monodisperse polystyrene (PS) microspheres with mean diameters of 211 and 418 nm (SD = 5–10 nm) were obtained from Microparticles GmbH as 5% (*w*/*v*) colloidal dispersions in deionized water (2.7–3.0% CV). Titanium(IV) bis(ammonium lactato) dihydroxide (TiBALDH, 50 wt.% in water), molybdenum disulfide dispersion (1 mg/mL in H_2_O, lateral size 50–1000 nm), Au NPs (5 nm, OD 1, citrate-stabilized, λ_max_ ≈ 510–521 nm), and Hellmanex^TM^ III were purchased from Sigma-Aldrich, St. Louis, MO, USA. Ag NPs (10 nm, 0.02 mg/mL in 2 mM sodium citrate, λ_max_ ≈ 390–400 nm) were sourced from Thermo Scientific Chemicals, Waltham, MA, USA. All other reagents, including ethanol (absolute, 99.8%), acetone (ACS reagent, ≥99.5%), and hydrochloric acid (ACS reagent, fuming, ≥37%), were of analytical grade.

### 2.2. Materials Fabrication and Characterization

**Figure 1:**
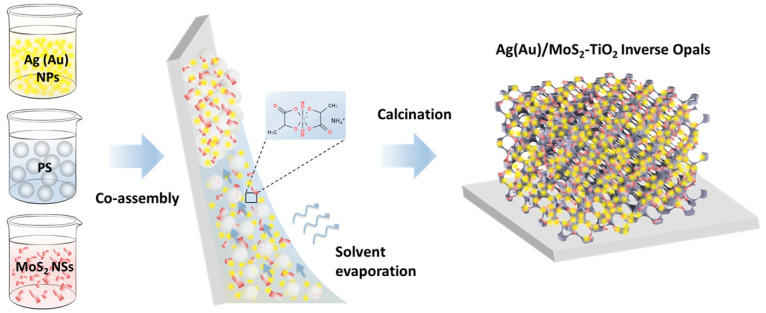
Schematic fabrication of Ag(Au)/MoS_2_–TiO_2_ IO films.

Plasmonic–photonic Ag(Au)/MoS_2_–TiO_2_ IO films were synthesized via evaporation-induced co-assembly of monodisperse PS spheres (211 and 418 nm in diameter) with a titania precursor mixture (0.25 mL TiBALDH, 0.5 mL 0.1 M HCl, and 1 mL ethanol), MoS_2_ NS dispersion, and citrate-stabilized suspensions of 10 nm Ag or 5 nm Au NPs (Figure 1). The smaller PS spheres (211 nm) were chosen based on prior results demonstrating optimal photocatalytic activity in unmodified TiBALDH-derived TiO_2_ IO films, owing to the spectral overlap of slow-photon modes near the red PBG edge with the anatase TiO_2_ absorption edge [60].

In contrast, the larger PS spheres (418 nm) were selected to maximize light harvesting in MoS_2_–TiO_2_ composites, where the photonic band gap aligns between the two prominent excitonic absorption peaks of MoS_2_ NSs [43]. To improve uniformity and co-assembly compatibility, cascade centrifugation was applied to the commercial MoS_2_ dispersion to narrow the lateral size distribution of the nanosheets and minimize aggregation, which otherwise hinders the formation of well-ordered IO structures [43]. Following probe sonication, the initial MoS_2_ dispersion was subjected to a stepwise centrifugation process. An initial low-speed spin at 1000 rpm was used to remove large aggregates, and the resulting supernatant was subsequently centrifuged at increasing speeds up to 8000 rpm. This gradual process reduced both the concentration and average size of MoS_2_ NS aggregates. Aliquots of 0.05 mL and 0.15 mL from the refined MoS_2_ dispersions were then used to partially replace Milli-Q water in 211 nm and 418 nm PS sphere suspensions, respectively. These suspensions were diluted to 0.125 wt.% and treated with ultrasound to ensure homogeneity before being mixed with 0.07 mL of a freshly prepared titania precursor. Plasmonic NPs (Ag or Au) were introduced into the mixture using an optimized addition volume of 0.2 mL from their respective colloidal dispersions. Clean fluorine-doped tin oxide (FTO) substrates (2.2 mm thick, 7 Ω/sq surface resistivity, Sigma Aldrich) or plain glass slides were then immersed vertically into 10 mL of the prepared colloidal mixtures. Solvent evaporation was carried out at 60 °C, allowing self-assembly of the PS opal template filled with the mixed precursor. The resulting dry composite films were calcined in air at 400 °C for 2 h (ramp rate 1 °C/min) to remove the PS spheres and crystallize the inverse opal TiO_2_ framework containing embedded MoS_2_ NSs and Ag/Au NPs. The plasmonic modified MoS_2_-TiO_2_ PC films were designated as Ag/Au 0.2-PCYYY-MoS_2_ 0.ZZ, with 0.2 and 0.ZZ representing the volumes (in mL) of the Ag/Au and MoS_2_ suspensions, respectively, while YYY denotes the diameter of the templating PS microspheres. Reference TiO_2_ IO films, namely PC211 and PC418, prepared from 211 and 418 nm PS microspheres as well as Ag/Au 0.2-PC211 and Ag/Au 0.2-PC418 IO films, were also prepared by the same method for comparison purposes.

The morphology and phase composition of the films were characterized using scanning electron microscopy (SEM) equipped with an energy-dispersive X-ray (EDX) analyzer, as well as an FEI Talos F200i field-emission scanning/transmission electron microscope (S/TEM) (Thermo Fisher Scientific Inc., Waltham, MA, USA) coupled with a windowless energy-dispersive spectroscopy (EDS) microanalyzer. The structural properties were investigated by X-ray powder diffraction (XRD) by a SmartLab Rigaku θ/θ Bragg–Brentano diffractometer (Rigaku, Tokyo, Japan), using a pyrolytic graphite monochromator and Cu Kα radiation. The continuous step-scanning mode was used at 0.03° steps with 11 s/step. The phase composition was further studied by micro-Raman spectroscopy using a confocal Raman microscope (LabRAM Soleil™, Horiba Scientific, Longjumeau, France) with 532 nm laser excitation, focused on the films’ surface using a 100× (NA = 0.9) objective. Optical characterization was conducted via diffuse and specular reflectance measurements using a Cary60 UV-Vis spectrophotometer (Agilent, Santa Clara, CA, USA), equipped with a fiber-optic diffuse reflectance probe and a 15° specular reflectance accessory. A Halon reference and a UV-enhanced aluminum mirror were employed for background calibration in the respective configurations. Ultraviolet photoelectron spectroscopy (UPS) was performed using He I excitation (21.22 eV photon energy). The work function (WF), referenced to the vacuum level, was calculated by subtracting the secondary electron cut-off energy from the incident photon energy. The cut-off was determined by extrapolating the linear portion of the low-binding energy edge of the spectrum to its intersection with the baseline. Similarly, the valence band maximum (VBM) position relative to the Fermi level (EF) was estimated from the difference E_F_-E_VBM_, obtained by extending the linear region of the valence band edge near E_F_ to intersect with the baseline of the spectrum.

### 2.3. Photocatalytic and Photoelectrochemical Evaluation

The photocatalytic activity of the Ag(Au)/MoS_2_–TiO_2_ IO films was initially evaluated via the visible-light-induced degradation of salicylic acid (SA) as a model pollutant [60]. SA was selected as it is a recalcitrant, colorless water contaminant, which, in contrast to dye pollutants, absorbs in the UV range, well below the PBG of the IO films and thus avoids slow photon contributions by spectral overlap with the target molecule absorption. The photodegradation tests were carried out at acidic pH = 3 that assists the chemisorption of SA molecules on titania and favors direct oxidation by valence band holes [60]. PC films (1 cm^2^) were placed horizontally in vials containing 4 mL of a 30 μM aqueous SA solution (pH = 3) and stirred in the dark for 60 min to establish adsorption–desorption equilibrium. A 150 W Xenon lamp equipped with a 305 nm long-pass filter and a heat-reflective mirror was used as the illumination source. For visible-light-only irradiation, an additional 400 nm long-pass filter was applied. The incident beam was directed onto the film using a UV-enhanced Al mirror at an intensity of 70 mW/cm^2^. Aliquots (0.5 mL) were periodically collected and analyzed using a UV-Vis spectrophotometer with a 10 mm quartz microcell. All photocatalytic tests were conducted in triplicate, and the mean kinetic constants were reported with standard errors.

Photoelectrochemical measurements were carried out in a standard three-electrode configuration using a CS350 potentiostat/galvanostat (Corrtest Instruments, Wuhan, China). The IO films on FTO substrates (4 cm^2^) served as working electrodes, with a Pt foil as the counter electrode and Ag/AgCl as the reference electrode. The electrolyte was an aqueous 0.5 M NaHCO_3_ solution. Visible light illumination (90 mW/cm^2^) was provided by a 300 W Xe lamp combined with a 400 nm long-pass filter. Electrochemical impedance spectroscopy (EIS) was carried out at open-circuit voltage over the 10^4^–10^−2^ Hz frequency range with a 10 mV AC amplitude. Mott–Schottky measurements were conducted at 500 Hz with a scan rate of 10 mV/s. Flat band potentials were determined from Mott–Schottky plots (1/C^2^ vs. applied potential) using the following equation:

(1) $$\frac{1}{C^{2}}=\frac{2}{eA^{2}\varepsilon \varepsilon_{0}N_{D}}\left(V-V_{fb}-\frac{kT}{e}\right),$$

where *C* is the space-charge capacitance, *e* is the elementary charge, *A* is the electrode area, *N*_D_ is the donor density, *ε* is the permittivity of the semiconductor, *ε*_0_ is the vacuum permittivity, *V*_fb_ is the flat band potential, *T* is the temperature, and *k* is Boltzmann’s constant.

The photoelectrocatalytic degradation of TC was also examined using the same three-electrode configuration under visible light (300 W Xe lamp (Corrtest Instruments, Wuhan, China), 400 nm long-pass filter, 90 mW/cm^2^), in a 40 mL solution of 0.1 M NaHCO_3_ containing 20 mg/L TC. The solution was gently stirred during irradiation. Aliquots were withdrawn periodically for UV-Vis analysis to monitor TC degradation. All experiments were repeated in triplicate, and the reported rate constants include standard error estimates. Furthermore, TC degradation was investigated by high-performance liquid chromatography (HPLC) using a Shimadzu Prominence-i LC-2030C 3D Plus (Kyoto, Japan) HPLC-DAD equipped with a quaternary pump. Chromatographic separation was achieved with a ZORBAX eclipse plus C18 column (150 mm × 4.6 mm, 5 μm), provided by Agilent. The separation mobile phase consisted of Solution (A), an acetonitrile–methanol mixture (60:40), and Solution (B), formic acid 1% in isocratic conditions. The total run was 12 min and the retention time was 5.9 min for TC and 5.7 min for epi-TC, the unavoidable epimer of TC. The column temperature was maintained at 25 °C, the injection volume was 20 μL, and the flow rate was 1 mL/min. For the determination of samples, solutions were directly injected to the chromatographic system. The results were expressed as the sum of TC and epi-TC and they were quantified using a standard solution of 11.5 ppm.

## 3. Results

### 3.1. Structural and Optical Properties

**Figure 2:**
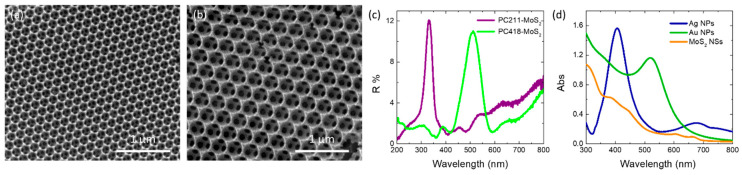
SEM images of the (**a**) PC211-MoS_2_ 0.05 and (**b**) PC418-MoS_2_ 0.15 co-assembled IO films, as well as (**c**) the corresponding Bragg reflection R% peaks in comparison to (**d**) the LSPR extinction of Ag, Au NP, and MoS_2_ NS suspensions.

Co-assembly of Ag and Au NP aqueous suspensions with polymer colloidal spheres and a hydrolyzed titania precursor was carried out for two selected MoS_2_–TiO_2_ IOs with distinct macropore sizes. This approach aimed to align the slow-photon spectral regions with the TiO_2_ absorption edge and the visible-light absorption of MoS_2_, respectively. Figure 2a,b display SEM images of the PC211-MoS_2_ 0.05 and PC418-MoS_2_ 0.15 films, fabricated using PS spheres of 211 and 418 nm in diameter. Both samples exhibit well-ordered, periodic IO architectures with interconnected macropores through smaller ones that appear at the contact points of adjacent PS spheres after calcination. The average macropore diameters that were determined for the two IOs were approximately 140 and 250 nm, respectively. Specular reflectance (R%) measurements at a 15° angle of incidence revealed distinct Bragg reflections, indicative of PBG formation, centered at 332 nm for PC211-MoS_2_ 0.05 and 512 nm for PC418-MoS_2_ 0.15 (Figure 2c).

The PBG positions can be estimated using the modified Bragg’s law for first-order diffraction from the (111) planes of a face-centered cubic (*fcc*) IO structure [60]:

(2) $$\lambda =2d_{111}\sqrt{n_{eff}^{2}-{sin}^{2}\theta },$$

where λ is the PBG wavelength, $d_{111}=\sqrt{2/3}D$ is the interplanar spacing, and D is the macropore diameter. The effective refractive index $n_{eff}$ is calculated as a volume-weighted average of the refractive indices of the colloidal spheres ${(n}_{sphere})$ and solid matrix $\left(n_{solid}\right)$ consisting primarily of TiO_2_, following

(3) $$n_{eff}^{2}=n_{sphere}^{2}f+n_{solid}^{2}\left(1-f\right)$$

Here, f is the volume filling fraction of the spheres ( f=0.74 for close-packed *fcc* lattices), and θ is the angle of incidence relative to the [111] direction.

By applying Equation (2) to the experimental R% peak positions at θ=15° and using the measured diameters, the effective refractive indices and the corresponding void fractions $\left(1-f\right)$ were determined in air (with $n_{solid}=n_{{TiO}_{2}}=2.55$ and $n_{air}=1.0$, Table 1). The calculated $\left(1-f\right)$ values were consistently lower than the theoretical 0.26, indicating incomplete filling of the IO lattice. Notably, this effect was less pronounced in samples with smaller macropores, in agreement with previous studies on co-assembled TiO_2_ IOs [31], which reported increased surface area and mesopore volume related to enhanced skeletal wall porosity. Furthermore, using the determined void fractions and a refractive index of $n_{H_{2}O}=1.33$, the PBGs were recalculated for aqueous environments, where photocatalytic reactions occur, shifting to 381 nm and 622 nm for the PC211-MoS_2_ 0.05 and PC418-MoS_2_ 0.15 films, respectively (Table 1).

**Figure 3:**
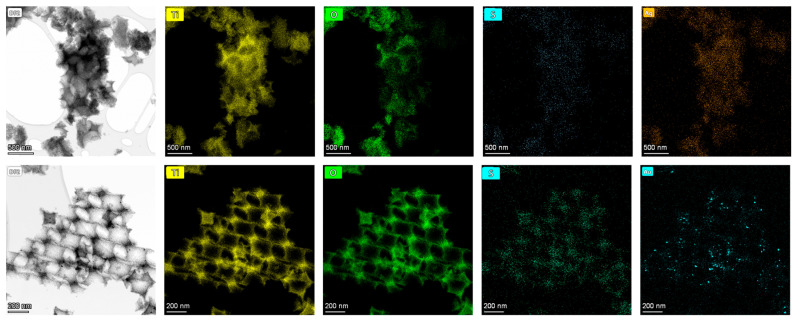
TEM images of (**upper row**) Ag 0.2-PC418-MoS_2_ 0.15 and (**bottom row**) Au 0.2-PC418-MoS_2_ 0.15 samples and the corresponding EDX elemental maps for Ti, O, S, and Ag/Au species.

The smaller-diameter IOs were selected for plasmonic modification because the unmodified TiO_2_ PC211 films exhibited the highest photocatalytic activity in SA degradation, attributed to the spectral overlap of red-edge slow photons with the anatase TiO_2_ absorption edge [60]. This spectral match could be further enhanced by the close proximity of the Ag NPs’ LSPR and the enhanced MoS_2_ absorption around 400 nm (Figure 2d). Larger-diameter PC418 IOs were also chosen, as they showed superior light-trapping properties, with the PBG positioned between the MoS_2_ excitonic absorption peaks at approximately 605 and 664 nm (Figure 2d), leading to the highest photocatalytic degradation rates for SA pollutants by co-assembled MoS_2_-TiO_2_ IO films under visible light [43]. TEM imaging confirmed that simultaneous incorporation of plasmonic NPs and MoS_2_ NSs into the PC418-MoS_2_ 0.15 films did not disrupt the IO structure after calcination (Figure 3), while EDX elemental mapping revealed uniform distributions of Mo, S, and Ag/Au species within the TiO_2_ skeleton.

**Figure 4:**
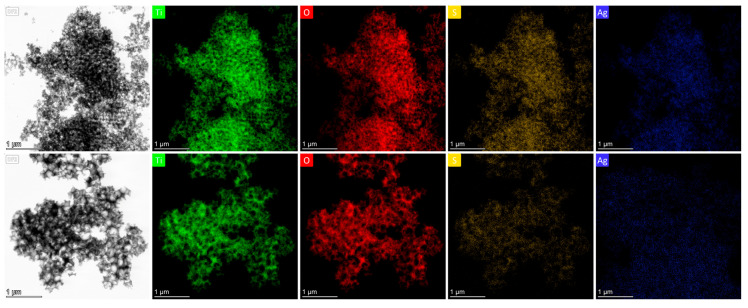
TEM images and the corresponding EDX elemental maps for Ti, O, S, and Ag species for (**upper row**) Ag 0.2-PC211-MoS_2_ 0.05 and (**bottom row**) Ag 0.2-PC418-MoS_2_ 0.15 IO samples.

Further TEM analysis was performed on samples prepared by 211 nm PS spheres (Figure 4) to verify whether the presence of Ag/Au NPs and MoS_2_ NSs impacted the IO structural integrity at smaller pore sizes. TEM images confirmed successful IO formation, although a lower MoS_2_ NS concentration was required during synthesis to preserve periodicity, due to the denser skeleton (higher filling fraction) of PC211 films. EDX mapping showed uniform Ag NP distribution in both films. However, quantitative EDX analysis indicated that the Ag 0.2-PC211-MoS_2_ 0.05 films accommodated significantly higher Ag NP loading compared to Ag 0.2-PC418-MoS_2_ 0.15 (Table 2), likely due to the increased surface area associated with smaller macropores and a higher density of skeletal interfaces.

**Table 2:** EDX analysis for the Ag-modified PC films.

Z	Element	Family	Atomic Fraction (%)	Atomic Error (%)	Mass Fraction (%)	Mass Error (%)
Ag 0.2-PC211						
8	O	K	66.25	3.78	39.60	4.04
22	Ti	K	33.71	3.78	60.30	4.05
47	Ag	L	0.04	0.01	0.10	0.02
Ag 0.2-PC211-MoS_2_ 0.05						
8	O	K	66.05	3.60	39.24	4.02
16	S	K	0.45	0.09	0.56	0.12
22	Ti	K	33.20	3.63	59.2	4.14
42	Mo	K	0.20	0.02	0.60	0.06
47	Ag	L	0.10	0.01	0.40	0.06
Ag 0.2-PC418						
8	O	K	66.04	4.16	39.29	3.54
22	Ti	K	33.94	4.17	60.65	3.55
47	Ag	L	0.02	0.00	0.06	0.01
Ag 0.2-PC418-MoS_2_ 0.15						
8	O	K	64.87	3.79	38.18	3.93
16	S	K	0.47	0.09	0.55	0.12
22	Ti	K	34.43	3.82	60.54	4.03
42	Mo	K	0.20	0.01	0.62	0.06
47	Ag	L	0.03	0.00	0.11	0.02

**Figure 5:**
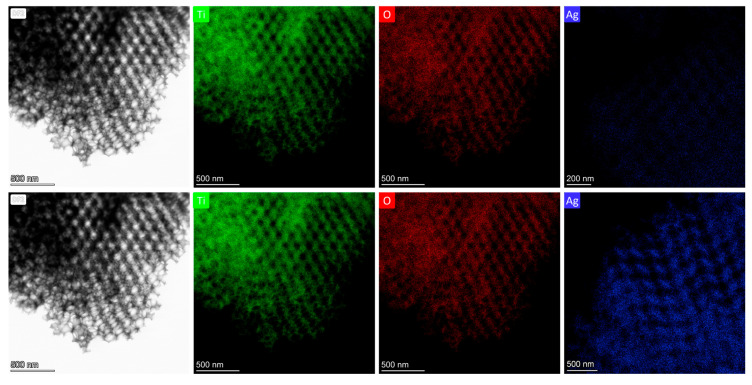
TEM images and the corresponding EDX elemental maps and spectra for Ti, O, and Ag species for (**upper row**) Ag 0.2-PC211 and (**bottom row**) Ag 0.2-PC418 IO films.

Comparative TEM and EDX analyses of reference Ag 0.2-PC211 and Ag 0.2-PC418 films (without MoS_2_) further revealed a notably higher Ag content in the PC211 films (Figure 5), reflecting the higher volume filling fraction of PC211 IOs with the smaller macropores, which present increased surface area and mesoporosity and thus favor NP loading [31]. This effect was significantly amplified with the incorporation of MoS_2_ NSs, in line with recent BET measurements showing that the addition MoS_2_ NSs increases the mesoporosity of the nanocrystalline TiO_2_ IO skeleton [43].

**Figure 6:**
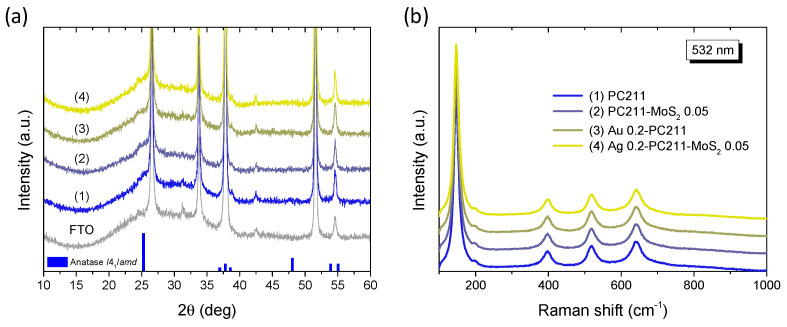
(**a**) XRD patterns and (**b**) Raman spectra at 532 nm of (1) Ag(Au) 0.2-PC211-MoS_2_ 0.05 compared to the pristine PC211 and PC211-MoS_2_ 0.05 IO films. The XRD patterns of the FTO substrate and the anatase TiO_2_ (JCPDS No. 21-1272) are also shown in (**a**).

XRD measurements on the Ag(Au) 0.2-PC211-MoS_2_ 0.05 IO films in comparison to their constituents (Figure 6a) did not show any distinct diffraction peaks, different from those of the FTO substrate. A featureless hump could be only traced at ~25°, which can be related to the excessively broadened (101) diffraction peak of the anatase TiO_2_ (space group *I*4_1_/*amd*) due to the small size of the anatase NPs (below ca. 10 nm) that form in co-assembled TiO_2_ IOs using the TiBALDH precursor [33,43]. In addition, no observable contribution could be detected from either the Ag(Au) NPs or the MoS_2_ NSs, because of their low content. This was verified by the corresponding Raman spectra (Figure 6b), where the characteristic Raman-active modes of anatase TiO_2_ were observed at 147 (E_g_), 199 (E_g_), 398 (B_1g_), 518 (A_1g_ + B_1g_), and 640 cm^−1^ (E_g_) with no signal from the characteristic Raman vibrations of MoS_2_ NSs, similar to the XRD results. All anatase Raman bands showed appreciable shifts and broadening with respect to bulk anatase [61], especially the most intense low frequency E_g_ mode that shifted to 147 cm^−1^ with a full width at half maximum (FWHM) of 16 cm^−1^. This effect can be related to the formation of small anatase NPs, which leads to the breakdown of the *q* = 0 selection rule for Raman scattering [61]. Specifically, using the E_g_ frequency and FWHM vs. NP size correlation curves [61], the formation of 8–9 nm anatase NPs can be predicted for the PC211 films, in agreement with previous reports on TiO_2_ IO films using TiBALDH [62].

**Figure 7:**
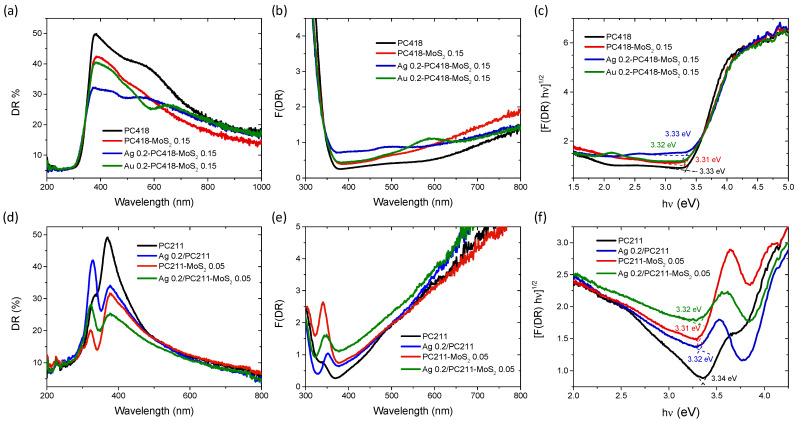
(**a**,**d**) Diffuse reflectance (DR%), (**b**,**e**) Kubelka–Munk absorbance spectra (F(DR)) and (**c**,**f**) the corresponding (indirect band gap) Tauc plots for the (**upper row**) Ag (Au) 0.2-PC418-MoS_2_ 0.15 IO films compared to the unmodified ones, as well as (**bottom row**) the Ag 0.2-PC211-MoS_2_ 0.05 and reference IO films.

The optical properties of the IO films were analyzed by diffuse reflectance (DR%) UV-Vis spectroscopy (Figure 7). Comparing the DR% spectra of the reference PC418 and PC418-MoS_2_ 0.15 samples with those of the plasmon-modified counterparts, a distinct decrease in reflectance was observed, appearing as local minima around 490 nm and 586 nm for the Ag- and Au-containing samples, respectively. These minima correspond closely to the LSPR absorption regions of Ag and Au NPS, though slightly red-shifted, likely due to aggregation effects of the plasmonic NPs [63].

Furthermore, the DR% of the Ag 0.2-PC418-MoS_2_ 0.15 films exhibited a significant reduction at wavelengths beyond 400 nm, above the anatase absorption edge, compared to the unmodified PC418-MoS_2_ 0.15 film. In contrast, the DR% decrease for the Au 0.2-PC418-MoS_2_ 0.15 film was confined to a narrower spectral range, reflecting the different spectral overlap of the LSPR with the DR% of the unmodified PC418 IO support. These variations were also observed in the Kubelka–Munk absorbance spectra (F(DR)) (Figure 7b), whereas only a slight decrease in the indirect band gap of anatase TiO_2_, reaching 2 meV, was derived for the Ag (Au) 0.2-PC418-MoS_2_ 0.15 IO films from the corresponding Tauc plots (Figure 7c). For the PC211 IO films, the diffuse reflectance of TiO_2_ dropped markedly due to the absorbance of the Ag NPs’ LSPR and/or MoS_2_ NSs around its band gap edge (Figure 2d), resulting in the clearer appearance of the IO skeletal Bragg reflection, which was barely discernible in the unmodified PC211 IO film. The synergistic absorbance of Ag NPs and MoS_2_ NSs accordingly produced the most pronounced DR% decrease at around 400 nm for the Ag 0.2-PC211-MoS_2_ 0.05 photonic films. These effects were further reflected in the corresponding F(DR) absorbance spectra (Figure 7e), while the derived Tauc plots indicated slight variations of the anatase band gap for the Ag 0.2-PC211-MoS_2_ 0.05 and the constituent IO films (Figure 7e), similar to the PC418 ones.

### 3.2. Photocatalytic Evaluation

**Figure 8:**
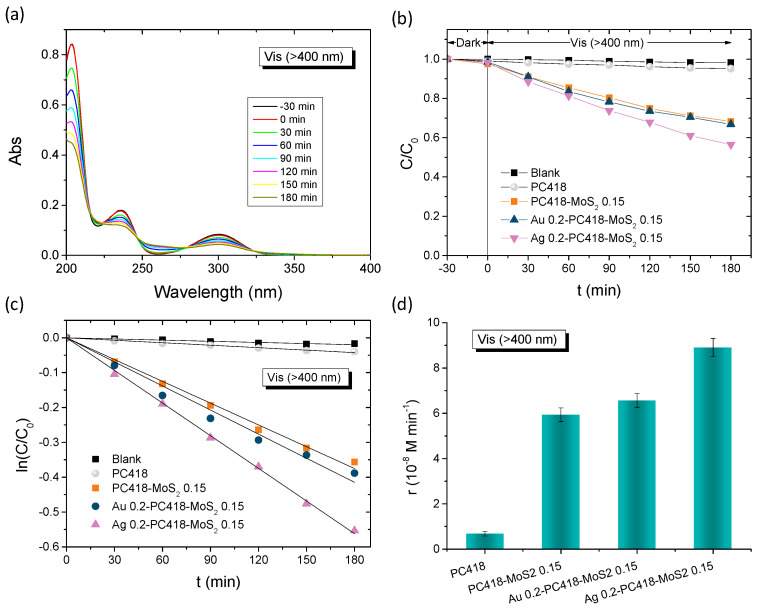
(**a**) Time dependence of SA absorbance spectra in the presence of Ag 0.2-PC418-MoS_2_ 0.15. (**b**,**c**) SA photodegradation kinetics and (**d**) reaction rates for the Au and Ag modified PC418-MoS_2_ 0.15 films in comparison to the reference ones under visible-light irradiation.

The plasmonic modified MoS_2_-TiO_2_ PC films were initially evaluated on the degradation of SA as model pharmaceutical pollutant under visible-light irradiation (*λ* > 400 nm) (Figure 8). Control experiments in the absence of films as well as in the presence of the pristine PC418 IO films showed negligible SA degradation under visible light (Figure 8b). In contrast, visible light illumination after dark adsorption in the presence of the IO films led to a continuous decrease in SA concentration (*C*), monitored spectrophotometrically via the SA absorption band at 300 nm (Figure 8a). The ln(*C*/*C*_0_) vs. time (*t*) plots, where *C*_0_ is the initial SA concentration after dark adsorption, exhibited linear behavior, indicating pseudo-first-order kinetics (Figure 8c). The kinetic constants (*k*, min^−1^) were derived from the slopes of these linear fits. Plasmonic modification enhanced the degradation rates, with *k* increasing by 50% for Ag 0.2-PC418-MoS_2_ 0.15, while only a modest ~10% improvement was observed for Au 0.2-PC418-MoS_2_ 0.15 (Figure 8d). The weak improvement of SA degradation by the Au-modified MoS_2_-TiO_2_ IO films, which are preferable due their higher stability, could be mainly related to the spectral mismatch of LSPR with the composite semiconductor absorption that precludes near-field amplification effects from the strongly absorbing 5 nm Au NPs [58] as well as the non-optimal spectral overlap of the observed PBGs (Table 1) with Au plasmonic absorption leading to weak enhancement of the hot electron injection mechanism that has been reported to be the key factor in Au-TiO_2_ IO photocatalysts [52,55,64,65].

**Figure 9:**
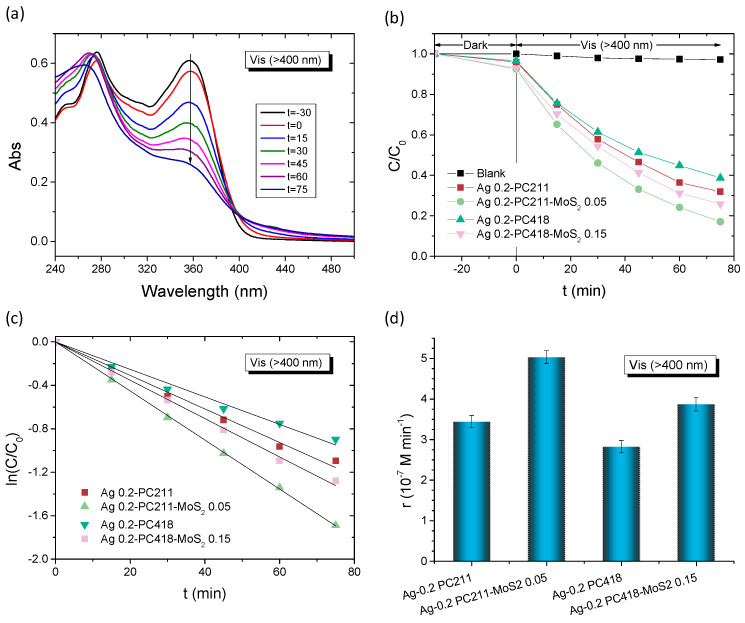
(**a**) Time dependence of TC absorbance in the presence of Ag 0.2-PC211. (**b**,**c**) TC photodegradation kinetics and (**d**) reaction rates for the Ag modified TiO_2_-MoS_2_ IO films in comparison to the Ag-modified TiO_2_ IOs under visible-light irradiation.

The best-performing Ag-modified MoS_2_-TiO_2_ PC films were subsequently selected for further photocatalytic evaluation of the degradation of TC under visible light for both IO diameters. Figure 9 summarizes the TC degradation results for the Ag-modified PC211-MoS_2_ 0.05 and PC418-MoS_2_ 0.15 films, compared to the corresponding Ag-modified PC211 and PC418 reference ones. After dark adsorption, the TC concentration, monitored via its 370 nm absorption band (Figure 9a), decreased steadily under illumination, again following first-order kinetics with *k* values extracted from the ln(*C*/*C*_0_) vs. *t* plots (Figure 9c). It should be noted that the highest TC dark adsorption was observed for Ag-modified MoS_2_-TiO_2_ PCs, reflecting the increase of surface area after the integration of MoS_2_ NSs in the TiO_2_ IO skeleton [43], whereas the incorporation of Ag NPs did not result in any appreciable variation in dark adsorption, because of the low content of plasmonic NPs. The visible-light reaction rates *r*_vis_ were then calculated as *r* = *k C*_0_, making them independent of dark adsorption variations. Notably, both Ag-modified MoS_2_-TiO_2_ PC211 and PC418 films exhibited significantly higher reaction rates compared to the corresponding Ag-modified PC211 and PC418 controls, confirming the essential role of MoS_2_ NSs in enhancing visible-light-driven photocatalysis. Interestingly, although the larger-diameter Ag-modified PC418-MoS_2_ 0.15 films, whose unmodified TiO_2_-MoS_2_ IO supports demonstrated optimal visible-light trapping and superior SA degradation [43], were outperformed by approximately 30% by the smaller-diameter Ag 0.2-PC211-MoS_2_ 0.05 films in TC degradation (Figure 9d).

This size-selective performance suggests that tuning the PBG closer to the TiO_2_ absorption edge and the LSPR absorption of Ag NPs, rather than matching the MoS_2_ visible-light absorption, is more effective for enhancing VLA photocatalytic activity. Assuming a PBG width corresponding to the full width at half maximum of the Bragg reflection R% peak of ≈40 nm (Figure 2c), the PBG centered at ~380 nm in water (Table 1) would extend between 360 and 400 nm. The associated red-edge slow photons, spanning roughly 20–30 nm above 400 nm, would closely match both Ag LSPR absorption and MoS_2_-TiO_2_ absorption. Thus, plasmonic effects, including local field enhancement for the strongly absorbing small-diameter Ag NPs [44] and/or hot electron injection from Ag NPs into the MoS_2_-TiO_2_ composite, together with electron transfer from MoS_2_ NSs [43], could be proposed as the primary contributors to the enhanced visible-light photocatalytic activity of the Ag-modified MoS_2_-TiO_2_ photonic crystals.

**Figure 10:**
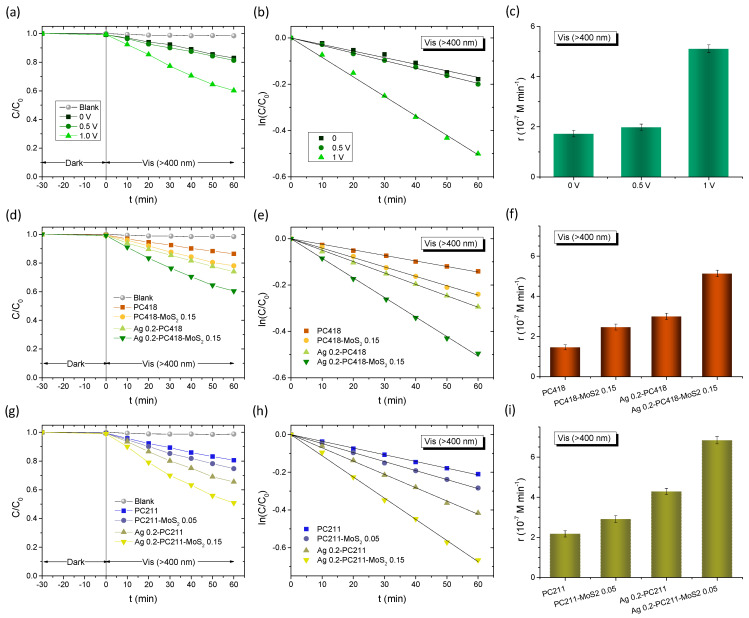
Visible-light-activated photoelectrocatalytic TC degradation kinetics and the corresponding reaction rates for (**a**–**c**) Ag 0.2-PC418-MoS_2_ 0.15 under no bias and under +0.5 and +1.0 V vs. Ag/AgCl, (**d**–**f**) Ag 0.2-PC418-MoS_2_ 0.15, and (**g**–**i**) Ag 0.2-PC211-MoS_2_ 0.05 photoelectrodes compared to the corresponding constituent IO films at +1.0 V vs. Ag/AgCl.

An advanced application of the Ag-modified MoS_2_-TiO_2_ PC films was further explored through photoelectrocatalytic degradation of TC using a three-electrode electrochemical setup under visible light (λ > 400 nm). The experiments employed an Ag/AgCl reference electrode and a 40 mL working solution—ten times larger than the volume used in the previous photocatalytic tests—consisting of 0.1 M NaHCO_3_ as supporting electrolyte containing 20 mg/L of TC under mild stirring. Initial tests were carried out on the Ag 0.2-PC418-MoS_2_ 0.15 photoelectrodes under three different conditions: without external bias and under applied potentials of +0.5 V and +1.0 V vs. Ag/AgCl (Figure 10a–c). A clear enhancement in TC degradation kinetics was observed with increasing applied potential, reaching optimal performance at +1.0 V vs. Ag/AgCl, indicative of more efficient electron–hole separation facilitated by the external bias.

It should be noted that in this case, no appreciable dark adsorption could be traced on the TC absorbance due to the relatively small size (4 cm^2^) of the photolectrode with respect to the reaction solution volume (40 mL).

Subsequent comparative evaluations were performed for both Ag 0.2-PC418-MoS_2_ 0.15 (Figure 10d–f) and Ag 0.2-PC211-MoS_2_ 0.05 (Figure 10g–i) IO films, alongside their corresponding reference samples, at an applied bias of +1.0 V vs. Ag/AgCl under visible light. In all cases, TC degradation followed first-order kinetics, with rate constants determined from linear fits of the ln(*C*/*C*_0_) vs. time plots. The incorporation of MoS_2_ NSs and Ag NPs into the TiO_2_ IO frameworks consistently resulted in enhanced reaction rates compared to the unmodified reference films, with the Ag-modified TiO_2_ photoelectrodes exhibiting higher performance. Moreover, the synergistic effect of combining MoS_2_ NSs and Ag NPs was evident, particularly for the Ag 0.2-PC211-MoS_2_ 0.05 films, which achieved reaction rates that surpassed those of the unmodified PC211-MoS_2_ and Ag 0.2-PC211 IO films by factors of 2.3 and 1.6, respectively (Figure 10i). These results strongly support the synergistic amplification mechanism involving slow-photon-enhanced light harvesting through spectral matching of the low-energy (red) edge of the inverse opal PBG with the Ag LSPR and the close-lying TiO_2_ absorption edge. This effect is further amplified by the contribution of the MoS_2_ NSs that enable visible-light activation and effective charge separation during the photoelectrocatalytic process. The obtained kinetic constant *k* for the Ag 0.2-PC211-MoS_2_ 0.05 IO films are comparable to the best values reported by visible-light-responsive photoelectrodes, including benchmark BiVO_4_ photoelectrodes (Table 3).

**Figure 11:**
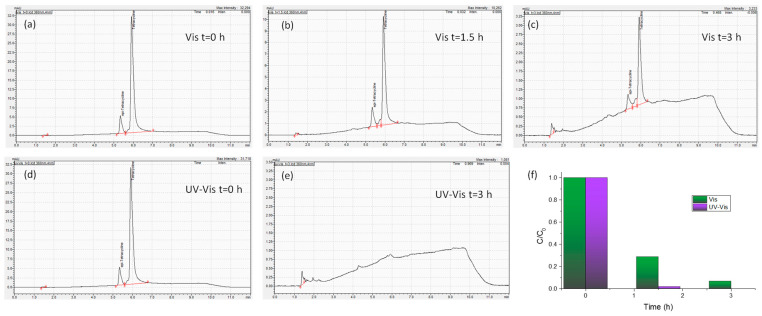
HPLC chromatograms of TC obtained during photoelectrocatalytic degradation experiments in the presence of Ag 0.2-PC211-MoS_2_ 0.05 IO films under visible light for (**a**) 0 h, (**b**) 1.5 h, and (**c**) 3 h as well as UV-Vis light for (**d**) 0 h and (**e**) 3 h at +1.0 V vs. Ag/AgCl. (**f**) The corresponding TC degradation kinetics up to 3 h under both illumination conditions.

Moreover, in order to validate the performance of the optimal Ag 0.2-PC211-MoS_2_ 0.05 IO photoanodes, the photoelectrocatalytic degradation of TC was also monitored by HPLC under visible and UV-Vis light for up to 3 h at +1.0 V vs. Ag/AgCl (Figure 11). The corresponding HPLC chromatograms show that TC degradation reaches 71.1% and 93.0% after 1.5 and 3 h under visible light, whereas much higher TC degradation levels of 98.0% and 100% could be achieved after 1.5 and 3 h under UV–visible light, supporting the high photocatalytic activity of the optimized Ag/MoS_2_-TiO_2_ IO photoanodes.

### 3.3. Band Alignment and Charge Separation

**Figure 12:**
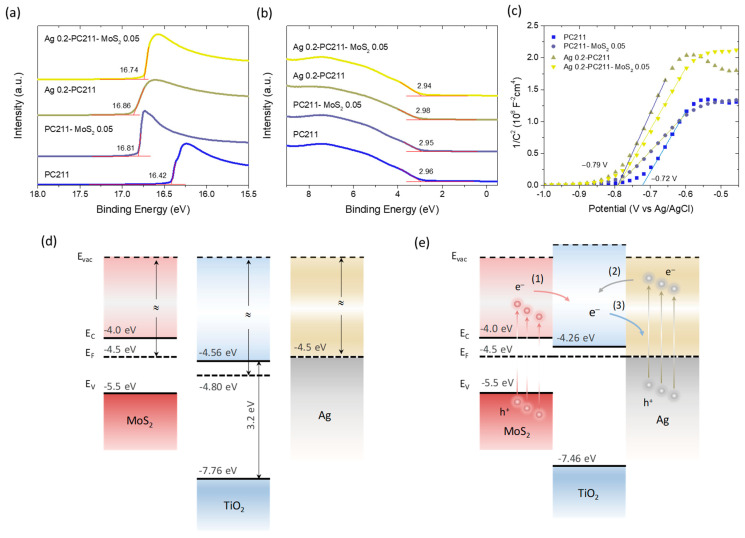
(**a**) Secondary electron cut-off spectra and (**b**) valence band UP spectra of Ag 0.2-PC211-MoS_2_ 0.05 and its constituent IO films, used to determine the WF and VBM positions relative to the Fermi level. (**c**) Mott–Schottky plots for the same films, showing flat band potential shifts after modification. (**d**) Schematic energy band alignment and (**e**) charge transfer pathways in the Ag-TiO_2_-MoS_2_ heterojunction system, before and after interfacial contact and E_F_ equilibration: (1) electron transfer from MoS_2_ to TiO_2_, (2) hot electron injection to TiO_2_ via the non-radiative LSPR decay of Ag NPs, and (3) electron transfer from TiO_2_ to Ag NPs acting as electron scavengers.

To investigate band alignment and charge separation at the photoelectrode/electrolyte interface, UPS measurements were performed on the Ag 0.2-PC211-MoS_2_ 0.05 IO film and its individual components. Figure 12a,b display the secondary electron cut-off and valence band regions, respectively, from which the WF and valence band maximum (VBM) positions relative to the Fermi level were extracted vs. the vacuum level. For the unmodified PC211 film, the WF and E_F_-E_VBM_ were determined as 4.80 eV and 2.96 eV, respectively, consistent with previously reported values for co-assembled TiO_2_ IOs [42]. Upon modification, the WF decreased by 0.40 eV (PC211-MoS_2_ 0.05), 0.45 eV (Ag 0.2-PC211), and 0.33 eV (Ag 0.2-PC211-MoS_2_ 0.05), while the VBM positions shifted only marginally (≈0.02 eV), indicating an upward shift of the TiO_2_ Fermi level due to heterojunction formation with MoS_2_ NSs or Ag NPs.

To further probe this behavior in the electrolyte environment, Mott–Schottky analysis was carried out to determine the flat band potentials (*V*_fb_) of the IO photoelectrodes (Figure 12c). For the unmodified PC211 film, *V*_fb_ was determined at −0.72 V vs. Ag/AgCl using the *x*-intercept of the best linear regression curve on the corresponding Mott–Schottky plot. All modified films (PC211-MoS_2_ 0.05, Ag 0.2-PC211, and Ag 0.2-PC211-MoS_2_ 0.05) showed similar negative shifts to −0.79 V vs. Ag/AgCl, supporting the E_F_ shift toward more negative potentials observed by UPS.

Based on these findings and values from the literature—WF~4.5 eV for multilayer MoS_2_ NSs on TiO_x_ [73], a band gap of 1.5 eV, and E_F_~0.5 eV below the conduction band minimum due to n-doping [74], as well as WF~4.5 eV for citrate-capped Ag NPs [75]—a tentative energy band diagram of the Ag-TiO_2_-MoS_2_ system can be proposed (Figure 12c,d). Since both MoS_2_ NSs and Ag NPs have higher WFs than TiO_2_, Fermi level equilibration after heterojunction formation induces an upward shift in the E_F_ of TiO_2_, consistent with both UPS and Mott–Schottky results. In the TiO_2_-MoS_2_ heterojunction, visible-light excitation of MoS_2_ can promote electron transfer to the TiO_2_ conduction band, enabling the formation of superoxide radicals, which are major reactive species in the degradation of TC under visible light [43].

For Ag–TiO_2_ interactions, two electron transfer pathways are plausible: (i) the injection of hot electrons into TiO_2_ from Ag NPs via the non-radiative decay of LSPR or (ii) electron transfer from TiO_2_ to Ag NPs, which can act as electron sinks to suppress recombination [9]. Given the improved photocatalytic performance of both TiO_2_-MoS_2_ and Ag-TiO_2_ IO films (Figure 10), visible-light sensitization of the TiO_2_ skeleton by both components appears highly likely. Moreover, the close spatial proximity of Ag NPs to the TiO_2_-MoS_2_ interface may lead to significant local field enhancement at LSPR wavelengths. Considering the spectral alignment among red-edge slow photons, Ag LSPR absorption, and the TiO_2_-MoS_2_ absorption edge, a synergistic radiative enhancement of charge carrier generation at the interface is proposed. This mechanism aligns with the higher performance observed for the Ag 0.2-PC211-MoS_2_ 0.05 IO films. On the other hand, in the case of Au/MoS_2_-TiO_2_ IO films, the higher work function of ~5.2 eV for bulk Au, which could be further increased by ~0.2 eV with citrate capping [75], would lead to a Schottky barrier at the Au-TiO_2_ interfaces with height of WF(Au)-χ(TiO_2_) = 0.84 eV, where χ is the electron affinity of 4.56 eV measured for the TiO_2_ PCs (Figure 12d). In the absence of appreciable spectral overlap of Au LSPR with TiO_2_-MoS_2_ absorption and the PBGs of the IO films to allow for local-field and slow-photon amplifications, the formation of a sizeable Schottky barrier could obstruct hot electron injection from the Au NPs to TiO_2_ under visible light, limiting the efficiency of visible light sensitization of the semiconductor system by plasmonic Au. It should be though noted that decreasing the size of Au NPs may cause a decrease in their WF [76,77], which may approach that of Ag NPs, leading to a similar band alignment. However, the absence of slow-photon effects and local-field enhancement may still impede photocatalytic performance.

## 4. Conclusions

In summary, heterostructured Ag (Au)/MoS_2_-TiO_2_ IO films were successfully fabricated via a one-step co-assembly approach, enabling the simultaneous incorporation of plasmonic NPs and MoS_2_ NSs into a highly ordered, porous TiO_2_ photonic framework. The resulting architectures act as efficient visible-light-responsive photoelectrocatalysts for TC degradation. Optical and structural characterization revealed that the synergistic interplay among slow-photon effects, plasmonic resonance, and semiconductor heterojunction formation plays a central role in enhancing visible light harvesting, charge separation, and catalytic efficiency. Among all tested systems, Ag/MoS_2_-TiO_2_ PC211 films showed superior photocatalytic and photoelectrocatalytic performance. This enhancement was attributed to optimal spectral alignment between the red-edge slow photons of the TiO_2_ IO PBG, the LSPR of Ag NPs, and the TiO_2_-MoS_2_ absorption edge. UPS and Mott–Schottky analysis confirmed that the incorporation of MoS_2_ NSs and Ag NPs leads to an upward shift in the Fermi level of TiO_2_, facilitating efficient electron transfer. The proposed energy band alignment suggests multiple contributing mechanisms: (i) visible-light excitation of MoS_2_ followed by electron injection into the TiO_2_ conduction band, (ii) plasmon-induced hot electron transfer from Ag NPs to TiO_2_, and (iii) local electromagnetic field enhancement at the TiO_2_-MoS_2_-Ag interface due to LSPR coupling. These effects are further amplified by slow-photon effects in the IO structure, which concentrate the optical field at these spectral regions and promote radiative enhancement near the interface. This work underscores the potential of integrating photonic, plasmonic, and semiconductor functionalities into a unified nanostructure for advanced water treatment applications and provides a route for designing multifunctional photocatalysts responsive to visible light.

## Figures and Tables

**Table 1:** Structural and optical properties of the MoS_2_-TiO_2_ IO films.

Film	*D* ^a^ (nm)	*λ*_exp_ (15°) ^b^ (Air)	*n*_eff_ (Air)	1 − *f*	*n*_eff_ (H_2_O)	*λ* (0°) ^c^ (Air)	*λ* (0°) ^c^ (H_2_O)
PC211-MoS2-0.05	140	332	1.48	0.21	1.67	337	381
PC418-MoS2-0.15	250	512	1.28	0.12	1.52	523	622

^a^ *D* = macropore diameter of the IO films determined by SEM; ^b^ *λ*_exp_ = PBG wavelength determined from the 15° R% spectra; ^c^ *λ* (0°) = PBG wavelength at 0° incidence.

**Table 3:** Performance comparison on the photoelectrocatalytic degradation of tetracycline antibiotics under visible-light irradiation.

Photoanode	Morphology	Pollutant/ Concentration	Irradiation Conditions	Kinetic Constant k (min^−1^)	Ref
WO_3_/Mo-BiVO_4_	Nanoplate arrays	TCH 10 mg/L	Xe 300 W, AM 1.5 1 V vs. SCE	0.0114	[66]
BiVO_4_	Nanowires	TCH 10 mg/L	Xe 300 W, AM 1.5 1 V vs. Ag/AgCl	0.0035	[67]
BiVO_4_/ZnO	Nanorods	TC 20 mg/L	Xe 300 W λ > 420 nm, 0.8 V	0.00867	[68]
ZnO/TiO_2_/Ag_2_Se	NP/Nanorods	OTC 5 mg/L	36 W blue LED, 17.3 mW/cm^2^, 1 V vs. Ag/AgCl	0.00821	[69]
N-doped carbon dots/Vo-rich TiO_2_	NCDs/Nanowire arrays	TC 50 mg/L	Xe 300 W, λ > 420 nm, 0.6 V vs. SCE	0.01911	[70]
FeOOH/1%Y-BiVO_4_	warm-like	TCH 0.2 mg/L	PLS-SXE 300 W AM 1.5, 0.7 V bias	0.00631	[71]
BiVO_4_/NiFe	nanoflowers	TC 20 mg/L	Xe 300 W 0.6 V vs. SCE	0.01281	[72]
Ag 0.2-PC211-MoS_2_ 0.05	Inverse opals	TC 20 mg/L	Xe 300 W, λ > 400 nm 90 mW/cm^2^, 1 V vs. Ag/AgCl	0.01125	This work

## Data Availability

The original contributions presented in this study are included in the article. Further inquiries can be directed to the corresponding author.
